# Detecting unannotated splicing events in short-read RNA-seq with SAMI, a UMI-aware Nextflow pipeline

**DOI:** 10.1093/bioinformatics/btag252

**Published:** 2026-05-21

**Authors:** Sylvain Mareschal, Valentin Wucher, Sarah Huet, Camille Léonce, Kaddour Chabane, Sandrine Hayette, Pierre-Paul Bringuier, Stéphane Pinson, Marc Barritault, Claire Bardel

**Affiliations:** Hospices Civils de Lyon, NGS-HCL plateform, Bioinformatics group, Lyon F-69000, France; Hospices Civils de Lyon, NGS-HCL plateform, Bioinformatics group, Lyon F-69000, France; Hospices Civils de Lyon, French Reference Center of Paraneoplastic Neurological Syndromes and Autoimmune Encephalitis, Lyon F-69000, France; Université Claude Bernard Lyon 1, Mechanisms in integrated life sciences Institute (MeLiS), INSERM U1314, CNRS UMR 5284, Lyon F-69008, France; Hospices Civils de Lyon, Hematology Department, Lyon F-69000, France; Université Claude Bernard Lyon 1, Centre International de Recherche en Infectiologie, INSERM U1111 / CNRS UMR5308, Team Lymphoma Immuno-Biology, Lyon F-69007, France; Université Claude Bernard Lyon 1, Institut des Sciences Pharmaceutiques et Biologiques, Lyon F-69008, France; Hospices Civils de Lyon, Department of Pathology, Lyon F-69000, France; Hospices Civils de Lyon, Hematology Department, Lyon F-69000, France; Hospices Civils de Lyon, Hematology Department, Lyon F-69000, France; Hospices Civils de Lyon, Department of Pathology, Lyon F-69000, France; Université Claude Bernard Lyon 1, Villeurbanne F-69100, France; Hospices Civils de Lyon, Genetics department, Lyon F-69000, France; Hospices Civils de Lyon, Department of Pathology, Lyon F-69000, France; Université Claude Bernard Lyon 1, Cancer Research Centre of Lyon (CRCL), INSERM 1052, CNRS 5286, Cancer Initiation and Tumoral Cell Identity Department, Lyon F-69008, France; Hospices Civils de Lyon, NGS-HCL plateform, Bioinformatics group, Lyon F-69000, France; Université Claude Bernard Lyon 1, Institut des Sciences Pharmaceutiques et Biologiques, Lyon F-69008, France; Hospices Civils de Lyon, Genetics department, Lyon F-69000, France; Université Claude Bernard Lyon 1, LBBE, CNRS UMR 5558, Villeurbanne F-69100, France

## Abstract

**Summary:**

Although RNA-sequencing has replaced microarrays for gene expression profiling over the past 15 years, its full potential for splicing analysis in clinical settings remains underexploited. Most available tools are tailored for large cohorts or known isoforms, limiting their applicability in routine diagnostics where non-recurring events must be identified in low-dimension datasets. We present SAMI (Splicing Analysis with Molecular Indexes), a fully-integrated UMI-aware pipeline designed to detect splicing events diverging from transcript annotations. Building upon the well-proven STAR aligner, SAMI introduces original post-processing of gaps and potential intron retention to maximize accuracy, along with clear graphical representations and tunable filtering stringency. The ability of SAMI and concurrent software to detect intragenic splicing aberrations and gene fusions was assessed, both on real data from a commercial control sample and simulated data generated with ASimulatoR.

**Availability and implementation:**

Nextflow pipeline and Singularity container recipe freely available under GPL 3 license at https://github.com/HCL-HUBL/SAMI

## 1 Introduction

Splicing analysis has been addressed by numerous tools in research contexts, where samples belong to predefined conditions with sufficient replicates: rMATS (Shen *et al.* 2014), SUPPA2 (Trincado *et al.* 2018), MAJIQ (Vaquero-Garcia *et al.* 2023) … Clinical diagnostic requires however, conclusions to be drawn on individual and uncharacterized samples, an issue that tools like SPOT (Ferraro *et al.* 2020), LeafCutterMD (Jenkinson *et al.* 2020) or FRASER 2 (Scheller *et al.* 2023) tried to address by looking for outliers in large cohorts of samples. These tools rely on statistical and/or denoising models that require high dimension data (both in terms of screened genes and “normal” samples to compare to), conditions which are hard to meet in facilities running disease-specific sequencing panels. While these methods have been shown to perform well in the identification of rare/unique splicing events, they are not suited for the identification of recurrent splicing events, even if they are deleterious.

SAMI was developed to address such a need, i.e. maximizing sensitivity in small panels where samples may be scarce or likely to harbor recurring events. As SpliceLauncher (Leman *et al.* 2020) is being used to analyze small panels in a diagnostic context by some french facilities, it served as a comparison point for SAMI performance. SAMI (Splicing Analysis with Molecular Indexes) was additionally designed to take full advantage of currently available sequencing panel kits, handling unique molecular indexes (UMIs) and stranded sequencing.

## 2 Implementation

**Figure 1:**
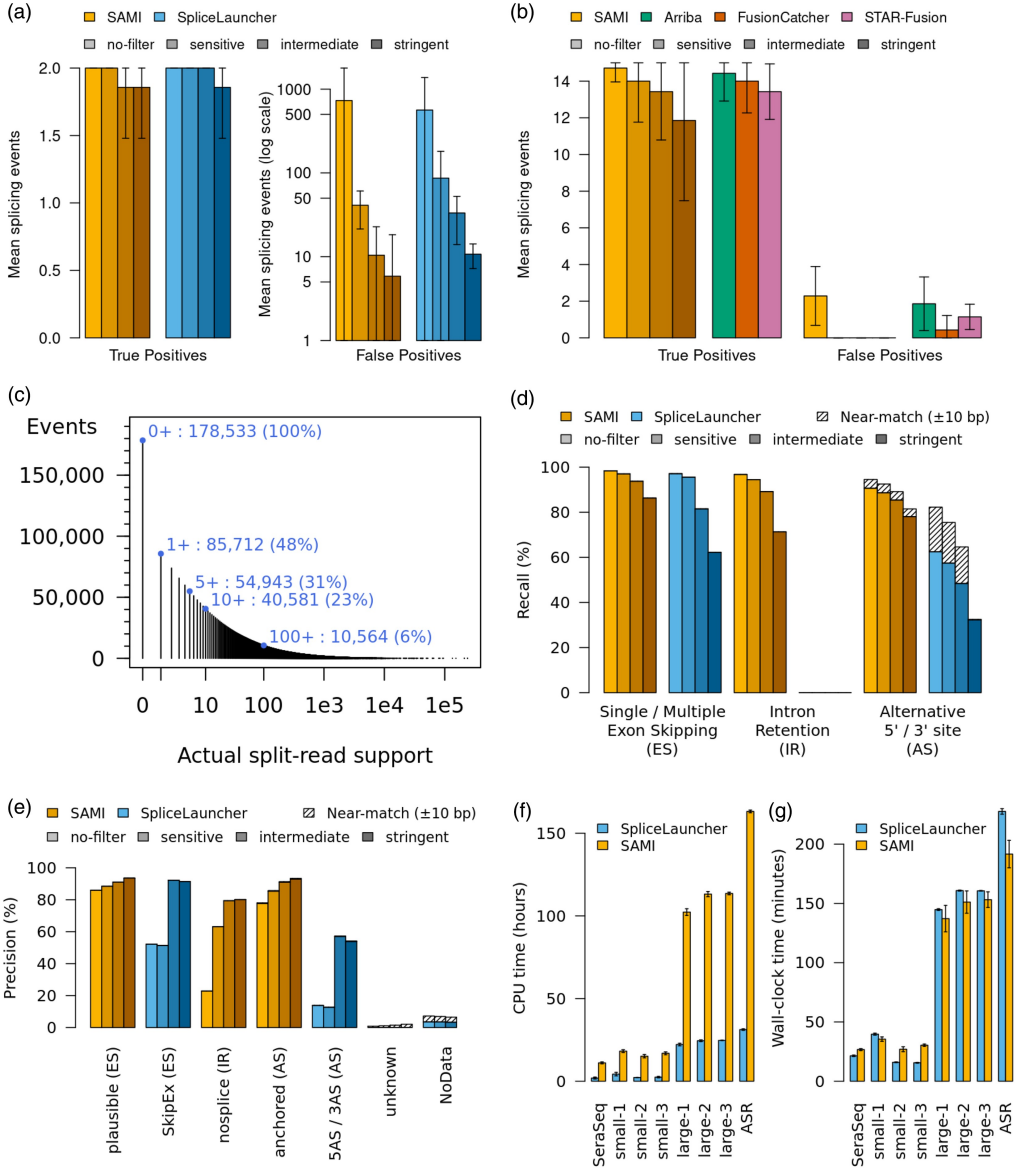
SAMI’s performances compared to SpliceLauncher and rnafusion. (a) Average of True (left panel) and False (right panel) positive splicing events detected on the seven Seraseq^®^ samples containing two expected splicing events (skips of MET exon 14 and EGFR exons 2 to 7) for SAMI (left) and SpliceLauncher (right). Four thresholds have been used: no-filter, sensitive, intermediate, and stringent (light shading). (b) Average of True (left panel) and False (right panel) positive fusion events detected on the seven Seraseq^®^ samples containing 15 expected fusion events for SAMI (left) and tools from rnafusion (right). For SAMI, four thresholds have been used: no-filter, sensitive, intermediate, and stringent. For rnafusion, the predictions of three tools are shown: Arriba, FusionCatcher, and STAR-Fusion. (c) Amount of splicing events generated by ASimulatoR supported by at least the number of split reads in X. (d) Proportion of splicing alterations created by ASimulatoR that were detected by SAMI and SpliceLauncher for each ASimulatoR event class and for various parameters of the two tools. Events found by a tool with slightly incorrect genomic coordinates (10 bp difference or less) were counted separately as near-matches. (e) Proportion of splicing alterations reported by SAMI and SpliceLauncher that were actually generated by ASimulatoR, split by event classes reported by each tool and for various parameters. Events found by a tool with slightly incorrect genomic coordinates (10 bp difference or less) were counted separately as near-matches. (f) CPU and (g) wall-clock computation time spent running SAMI or SpliceLauncher on 3 real-life runs of two RNA-seq panels of different sizes, on Seraseq^®^ data and on data generated by ASimulatoR (mean and standard deviation over five replicates of each).

SAMI is an integral Singularity-contained Nextflow (Di Tommaso *et al.* 2017) pipeline, processing raw FASTQ files directly from the sequencer (Fig. 1, available as supplementary data at *Bioinformatics* online). It covers usual RNA-seq processing steps such as adapter detection with AdapterRemoval (Schubert *et al.* 2016) and trimming with cutadapt (Martin (2011), two-pass alignment to the genome with STAR (Dobin *et al.* 2013) and multiple quality checks gathered with MultiQC (Ewels *et al.* 2016). These quality controls include FastQC (Andrews (2010), Picard’s CollectRnaSeqMetrics (https://broadinstitute.github.io/picard), featureCounts (Liao *et al.* 2014) and several custom controls.

SAMI takes full benefit from the two-pass alignment performed by STAR, which has been shown to improve the accuracy of splicing discovery (Veeneman *et al.* 2016). Splicing gaps are collected by STAR from each sample separately during the first pass and are injected into the reference genome to provide consistent gaps among samples during the second pass. SAMI further refines this gap collection by shifting novel junctions toward annotated splicing sites when multiple alignments are possible, enhancing accuracy significantly over alternative 5’/3’ splicing aberrations.

This two-pass approach also provides an opportunity to efficiently handle Unique Molecular Indexes (UMIs), which are now part of several commercial RNA-seq kits and allow for a more accurate deduplication than sequence-based approaches (Fu *et al.* 2018). In SAMI, mapping coordinates from the first pass are used by fgbio (Fennell and Homer (2016) to generate consensus reads from UMI clusters, which are aligned during the second pass. In the absence of UMIs, SAMI runs a classical two-pass STAR mapping.

Splicing analysis is performed by a collection of scripts integrated in SAMI. Evidence of splicing is collected from UMI-deduplicated BAM files by an HTSlib-based (Bonfield *et al.* 2021) C program, along with read pair orientation. Junctions are then annotated using Rgb (Mareschal *et al.* 2014) and the provided reference transcriptome, and classified according to the status of the two splicing sites involved:

- Annotated: both sites are annotated, and at least one annotated transcript holds it;
- Plausible: both sites are annotated, but no transcript exists with this junction;
- Anchored: only one site is annotated;
- Unknown: none of the sites is annotated.

This approach allows SAMI to detect both intra-gene splicing and gene fusion events, and particular care was taken with STAR parameters to achieve high sensitivity for both applications (Methods, available as supplementary data at *Bioinformatics* online).

To filter candidate events, SAMI provides two PSI (Percent Spliced In) values for each junction in each sample, reflecting how frequently it occurs among all splicing observed at the two involved splicing sites. ‘PSI’ values provided here are restricted to a single ‘junction’ in the context of a single splicing ‘site’, as opposed to tools like rMATS (Shen *et al.* 2014) which try to integrate multiple evidence sources into predefined splicing events. The support of a splice at a specific site ($\mathrm{read}s_{\mathrm{site}}$) is the amount of split reads evidencing a mapping gap starting or ending at this site.


$$PSI_{\mathrm{site},\mathrm{junction}}=\frac{\mathrm{read}s_{\mathrm{junction}}}{\sum reads_{\mathrm{site}}}$$

SAMI further extends this PSI-based approach by considering the absence of a splice at a site (as observed in intron retention or exon elongation) as an alternative during PSI computation. This is achieved by measuring the sequencing depth 3 bases inside the putative intron, i.e. after or before the considered splicing site, for all annotated and discovered splicing sites. These alternatives add two more classes for junctions returned by SAMI:

- No-splice: absence of splice at an annotated splicing site;
- Trivial: absence of splice at a de novo splicing site (inside an annotated intron or exon).

As final products, SAMI provides both filtered and unfiltered junction tables with extensive annotation, and graphical representations of each gene in each sample which evidences at least one filtered junction of interest (Fig. 2, available as supplementary data at *Bioinformatics* online).

## 3 Performances

Performances of SAMI and SpliceLauncher were assessed in two datasets with four levels of filter stringency: no filtering, sensitive, intermediate, and stringent (Methods, available as supplementary data at *Bioinformatics* online).

### 3.1 Seraseq^®^ commercial samples

The first dataset consisted of several replicates of a commercially available synthetic RNA sample (Seraseq^®^, LGC SeraCare, Milford, USA), containing two splicing events (skipping of exon 14 in *MET* and of exons 2 to 7 in *EGFR*) and 15 gene fusions of clinical interest (Table 1, available as supplementary data at *Bioinformatics* online). It was processed seven times in clinical conditions with a regular RNA quantity of 10 ng and six times with an increasing RNA quantity: 6.25, 10, 12.5, 25 ng, and twice with 50 ng. RNA capture was performed with a custom 18 kb panel; the resulting library was sequenced on a MiSeq sequencer (Methods, available as supplementary data at *Bioinformatics* online).

While SAMI and Splicelauncher both have a high mean number of false positive splicing events without filtering (731 and 562, respectively), this number is greatly reduced by using the sensitive filter (41 and 87 false positives, respectively, Fig. 1a). All expected events are found in all samples without filtering, but one event with low support is missed with an intermediate (SAMI) or a stringent (SpliceLauncher) filter. This can be explained by the different definitions of the filters for each tool. Moreover, some events labeled as false positives could be unannotated true events, thus falsely increasing the number of false positives.

Regarding gene fusion detection, SAMI was compared to the nf-core pipeline (Ewels *et al.* 2020) “rnafusion” version 3.0.2 (Renevey *et al.* 2024), which includes Arriba (Uhrig *et al.* 2021), FusionCatcher (Nicorici *et al.* 2014), and STAR-fusion (Haas *et al.* 2019), using fastp_trim and the default parameters. While all methods have similar performances in terms of true positives, SAMI didn’t detect any false positive when using a filter (Fig. 1b). On increasing quantity of Seraseq RNA, SAMI reached its recall plateau at 10 ng without a filter and at 25 ng with the most permissive filter, while the other methods did so at 10 ng (Fig. 3, available as supplementary data at *Bioinformatics* online).

### 3.2 Simulated dataset

To provide a broader overview of SAMI’s and SpliceLauncher’s capabilities regarding splicing, RNA-seq data with known aberrations was generated using ASimulatoR (Manz *et al.* 2021). FASTQ files were produced to simulate 16 million pairs of 100 bp reads per sample from MANE. Select transcripts of the RefSeq GRCh38 transcriptome. Thirty samples were produced through 10 simulations, each simulation expressing at various levels 8000 genes harboring each a single event of exon skipping, multiple-exon skipping, intron retention, alternative 5’ or 3’ splicing site (12.5% chance each), or no event (37.5% chance). The whole cohort was analyzed by SAMI and SpliceLauncher with default parameters and full RefSeq GRCh38 annotation.

As ASimulatoR synthetically expresses genes at a wide range of levels, 92 821 (52.0%) of the 178 533 simulated events were not supported by any generated split read and were thus impossible to detect (Fig. 1c). Focusing on 40 581 events supported by at least 10 simulated reads, SAMI’s recall with “intermediate” filtering was 93.8%, 89.1%, and 85.4% for exon skips (ES), intron retentions (IR) and alternative 5’/3’ sites (AS), respectively (Fig. 1d), with corresponding precision values of 91.1%, 79.4%, and 90.9% (Fig. 1e). Similar ES values were obtained with SpliceLauncher (81.5% recall and 92.2% precision), but recall was significantly lower on AS (48.4%), and IR is out of the tool’s scope. Up to 16.2% of AS events were not reported accurately by SpliceLauncher (shifted by 10 bp or less and improperly classified) but still present in the results, theoretically raising recall to 64.6% with “intermediate” filtering.

As opposed to SAMI, SpliceLauncher works with a single transcript definition per gene. In the results presented here, the transcripts used for the simulations were not indicated to SpliceLauncher.

### 3.3 Computation time

SAMI and SpliceLauncher ran on a dedicated computing node exposing 900 GB of RAM and 110 computing threads from two Intel^®^ Xeon^®^ Platinum 8352M CPUs to a SLURM instance. Computing time was assessed for 3 runs of a small MiSeq panel (16 × 0.8 million read pairs from 21 loci), 3 runs of a larger NextSeq panel (16 × 10 million read pairs from 165 genes), and a single run of ASimulatoR synthetic data (30 × 16 million read pairs from 8000 genes).

SpliceLauncher proved to be 4–6 times lighter than SAMI in terms of CPU time (Fig. 1f), but efficient parallelization in SAMI resulted in similar wall-clock durations (Fig. 1g). The higher computing intensivity of SAMI could be explained mainly by quality checks not performed by SpliceLauncher, more complex handling of UMIs, and different parameters for STAR alignment (Table 2, available as supplementary data at *Bioinformatics* online).

## 4 Clinical application

In clinical practice, skippings of *MET* exon 14 can be evidenced either by splicing-site mutations in DNA sequencing (Fujino *et al.* 2021) or directly by RNA sequencing. To illustrate SAMI’s capabilities in a clinical diagnostic context, we report results from both approaches (Methods, available as supplementary data at *Bioinformatics* online) for patients analyzed between 2024 and 2025 in our facility.

From a total of 1659 processed samples, 45 and 248 were not interpretable by DNA sequencing and RNA sequencing respectively (28 by both methods). In 1393 samples interpretable by both methods, 1385 samples (99.4%) were in agreement: 1349 negatives and 36 positives.

Only one potential false negative (0.07%) was identified, with a DNA mutation of unclear significance and limited evidence of skipping in RNA (PSI below 10%, case 45, Table 3, available as supplementary data at *Bioinformatics* online). Seven samples seemed positive in RNA only (0.5%), in which three DNA mutations initially missed out were actually found after manual examination of the BAM files (cases 37, 38, and 43) and two had low support (PSI below 15%, cases 39 and 41, Table 3, available as supplementary data at *Bioinformatics* online).

## 5 Conclusion

SAMI is a powerful tool for the detection of unannotated splicing events in disease-specific RNA-seq panels. Compared to SpliceLauncher, it offers equivalent performances in the detection of exon-skipping events significantly better ones on alternative 5’/3’ splicing sites thanks to original gap post-processing, and moreover, it detects intron retentions and gene fusions. Its implementation as a heavily parallelized, Singularity-contained Nextflow pipeline and optional compatibility with UMIs and stranded RNA-seq kits make it a portable and modern solution for processing RNA-seq data in a clinical context.

SAMI is a clinically adaptable tool that not only detects known splicing events but also uncovers novel aberrations with high precision in low-replicate scenarios—a key unmet need in translational genomics.

## Supplementary Material

btag252_Supplementary_Data

## Data Availability

The data underlying this article will be shared on reasonable request to the corresponding author.
