# Supplementary material for: Detecting unannotated splicing events in short-read RNA-seq with SAMI, a UMI-aware Nextflow pipeline
Source: Bioinformatics. 2026 May 21;42(6):btag252. doi: 10.1093/bioinformatics/btag252 (PMC13242923; doi:10.1093/bioinformatics/btag252)
Supplement: btag252_Supplementary_Data [file btag252_supplementary_data.pdf]

Detecting unannotated splicing events in short-read RNA-seq with SAMI, a UMI-aware Nextflow pipeline

March 27, 2026

Contents

1 Supplemental Methods **1**
1.1 SAMI . . . 1
1.2 STAR parameters . . . 1
1.3 SpliceLauncher . . . 2
1.4 ASimulatoR . . . 2
1.5 Seraseq® commercial sample . . . 2
1.6 RNA-sequencing panel . . . 3
1.7 DNA-sequencing of MET . . . 3

2 Supplemental Figures **3**
2.1 Supp Figure 1: Overview of SAMI's workflow . . . 3
2.2 Supp Figure 2: Example of plots generated by SAMI . . . 4
2.3 Supp Figure 3: True and False positive fusion events on increasing RNA concentration . . . 5

3 Supplemental Tables **6**
3.1 Supp Table 1: Events expected in the Seraseq® sample . . . 6
3.2 Supp Table 2: Detailed computation time . . . 6
3.3 Supp Table 3: DNA mutations of MET related to exon-skippings . . . 8

1 Supplemental Methods

1.1 SAMI

All analyzes were performed with SAMI version 2.1.0, using GCA_000001405.15_GRCh38_full_analysis_set reference genome and RefSeq annotation from 2024-08-23. The 4 profiles described in the article correspond to the following launch parameters:

```
no-filter:    --min.I=1  --min.PSI=0
sensitive:    --min.I=3  --min.PSI=0.01
intermediate: --min.I=5  --min.PSI=0.05
stringent:    --min.I=10 --min.PSI=0.1
```

Annotated events, i.e. "annotated" and "trivial" classes, have been ignored during analyzes.

1.2 STAR parameters

As SAMI's ability to detect splicing events heavily relies on read alignements provided by STAR [1], especially chimeric reads which are discarded with default parameters, specific care was taken for its parametrization. STAR alignment parameters implemented by STAR-fusion [2] (in a context of intergenic fusion detection) and SpliceLauncher [3] (in a context of intragenic splicing event detection) were systematically reviewed and combined to tune SAMI's parameters. The full list of retained parameters can be consulted in the `modules/STAR/align/main.nf` file of SAMI's GitHub repository (https://github.com/HCL-HUBL/SAMI), and covers many aspects like chimeric junction detection and scoring (`--chim...`), deactivation of the minimal distance between splicing sites (`--outSJfilterDistToOtherSJmin`) or merging of overlapping mates in pair-end sequencing (`--peOverlap...`).

1.3 SpliceLauncher

All analyzes were performed using a fork of https://github.com/LBGC-CFB/SpliceLauncher [3] from commit `f2f11113` (2022-07-01), adding minor adaptations to local computing infrastructure. Singularity recipe and annotation were built following the provided README on 2022-09-20. Unfortunately issues with latest versions of SpliceLauncher were not solved at the time of publication and prevented the benchmark to be performed with more recent versions.

For real-life data including Unique Molecular Indexes (UMIs), a deduplication step with CReaK from the Agilent Genomics NextGen Toolkit (AGeNT) version 3.1.2 was performed between separate calls to SpliceLauncher for alignment and counting, with the following arguments: `--consensus-mode="SINGLE" --remove-dup-mode -f --MBC-mismatch=1 --min-avg-MBC-qual=20 --min-avg-read-qual=20 -F -MS`.

The 4 profiles described in the article correspond to the following post-processing filters:

```
no-filter:    sample count >= 1
sensitive:    sample count >= 3
intermediate: sample count >= 5  & sample expr >= 0
stringent:    sample count >= 10 & sample expr >= 1
```

Annotated events, i.e. “Physio” types, have been ignored during analyzes.

1.4 ASimulatoR

RNA-seq data with known splicing events was generated with ASimulatoR [4] version 1.0.0 running in R version 4.0.4. Transcripts were extracted from `GCA_000001405.15_GRCh38_full_analysis_set` RefSeq annotation from 2024-08-23, excluding all scaffolds except chromosomes 1 to 21, X and Y. ASimulatoR pools exons from all annotated transcripts at the gene level, using chimeric transcripts never observed in the annotation as ”templates” and thus introducing unexpected splicing aberrations. To bypass this issue, only one transcript (the one selected by MANE) was used for each gene. `simulate_alternative_splicing()` was called 10 times with the following arguments:

```
event_probs = c(es=0.125, mes=0.125, ir=0.125, a3=0.125, a5=0.125)
multi_events_per_exon = FALSE
max_genes = 8000
num_reps = rep(1, 3)
meanmodel = TRUE
readlen = 100
paired = TRUE
seq_depth = 10e6
adapter_contamination = TRUE
pcr_rate = 0.3
distr = "empirical"
error_model = "illumina5"
bias = "none"
strand_specific = TRUE
gzip = TRUE
```

As event annotation and counts provided by ASimulatoR proved to be unreliable, split-reads supporting exon junctions were recounted from transcript coordinates provided by ASimulatoR in read names. Template and variable transcripts described in `splicing_variants.gtf` were systematically compared to identify novel junctions induced by exon skips (ES), multiple consecutive exon skips (MES), alternative 5’ (a5) and 3’ (a3) splicing sites. Similarly, the two expected “no-splice” events induced by each intron retention (IR) were inferred from these transcripts. Recall of SAMI ans SpliceLauncher was computed as the ability to retrieve these novel junctions, looking at genomic coordinates rather than transcript annotation.

1.5 Seraseq® commercial sample

18-plex Seraseq Fusion RNA Mix v4 reference standard sample was purchased from LGC Seracare (Milford, USA) and sequenced multiple times with the “small” RNA-seq panels described below. The two splicing events and 16 gene fusions expected in this control samples are described in Supp Table 2.

1.6 RNA-sequencing panel

RNA was extracted using the Maxwell® RSC RNA FFPE Kit (Promega, Reference AS1440) and the Maxwell® RSC Instrument (Promega, Catalog Number AS4500) from Formalin-Fixed, Paraffin-Embedded (FFPE) samples. According to manufacturer instructions, RNA libraries were prepared using the KAPA RNA HyperPrep Kit in combination with the KAPA Universal UMI Adapter with a sample input of 10 ng. The workflow involved pre-PCR for 18 cycles, followed by target enrichment using the KAPA HyperPETE LC Fusion Panel, an 18 kb capture target panel that includes 17 lung cancer fusion genes and 4 housekeeping genes as internal controls. Libraries were captured using the KAPA HyperPETE Reagent Kit and sequenced on an Illumina Miseq® System.

1.7 DNA-sequencing of MET

After selection of high tumor content zones, total nucleic acids were extracted using Maxwell® RSC RNA FFPE Kit (Promega, Reference AS1440) and the Maxwell® RSC Instrument (Promega, Catalog Number AS4500) without RNAse. *MET* splice site mutations were detected using a custom AmpliSeq library (Thermo Fisher Scientific, USA) with primers amplifying from c.2942-96 to c.2989 (5'splice site, NM_000245.4) and from c.3026 to c.3082+117 (3' splice site, NM_000245.4), analyzed with Torrent Suite Software 5.6. The complete list of DNA mutations of interest is provided as Supp Table 3.

2 Supplemental Figures

2.1 Supp Figure 1: Overview of SAMI's workflow

The main processes of SAMI are illustrated with blue boxes, sample-specific inputs and outputs with dotted lines and run-level ones with solid lines. Processes generating QC data aggregated in the final MultiQC reports are marked with a magnifying glass icon.

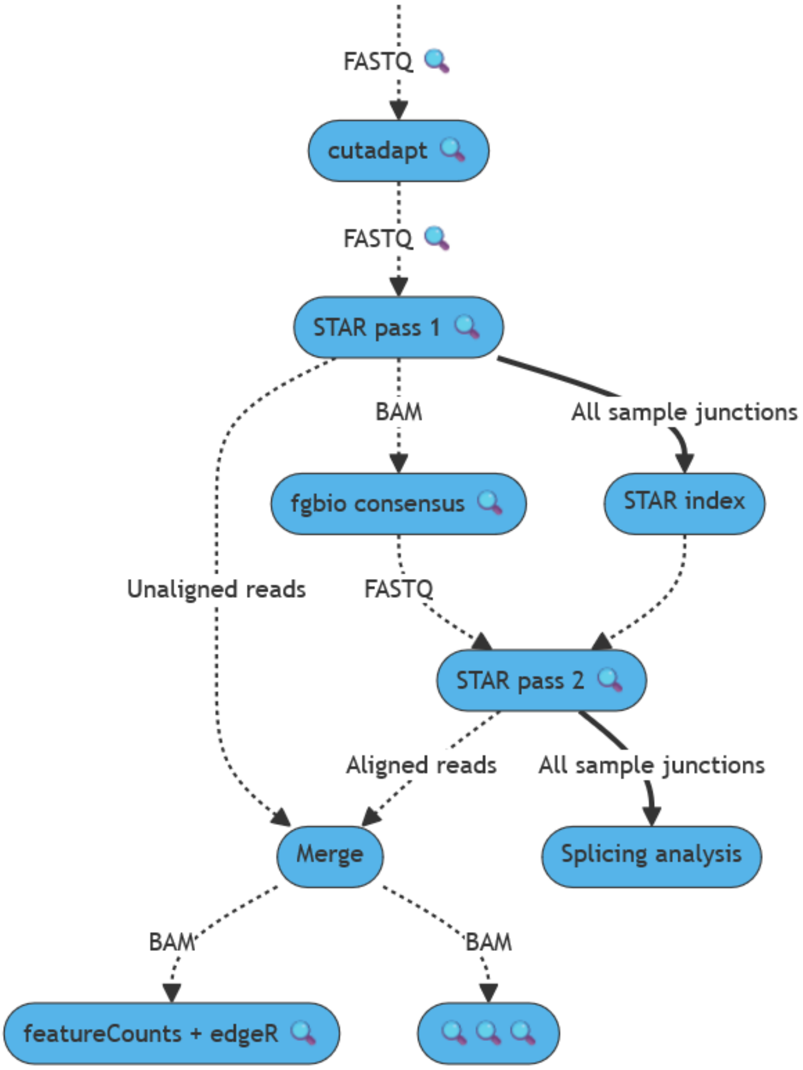

2.2 Supp Figure 2: Example of plots generated by SAMI

SAMI produces one plot for each gene (here *ENG*) and each sample harboring at least one event passing filters. The three transcripts described in the annotation for the considered gene are presented in the middle, with square boxes representing exons (numbered here in reverse order as *ENG* is transcribed from the reverse genomic strand). Exons are shaded according to the average sequencing depth observed in the sample, from lowest observed value in white to deepest coverage in black. Blue bridges on the top side illustrate the amount of split reads supporting annotated junctions, while colored bridges at the bottom represent aberrant splicing observed in the sample.

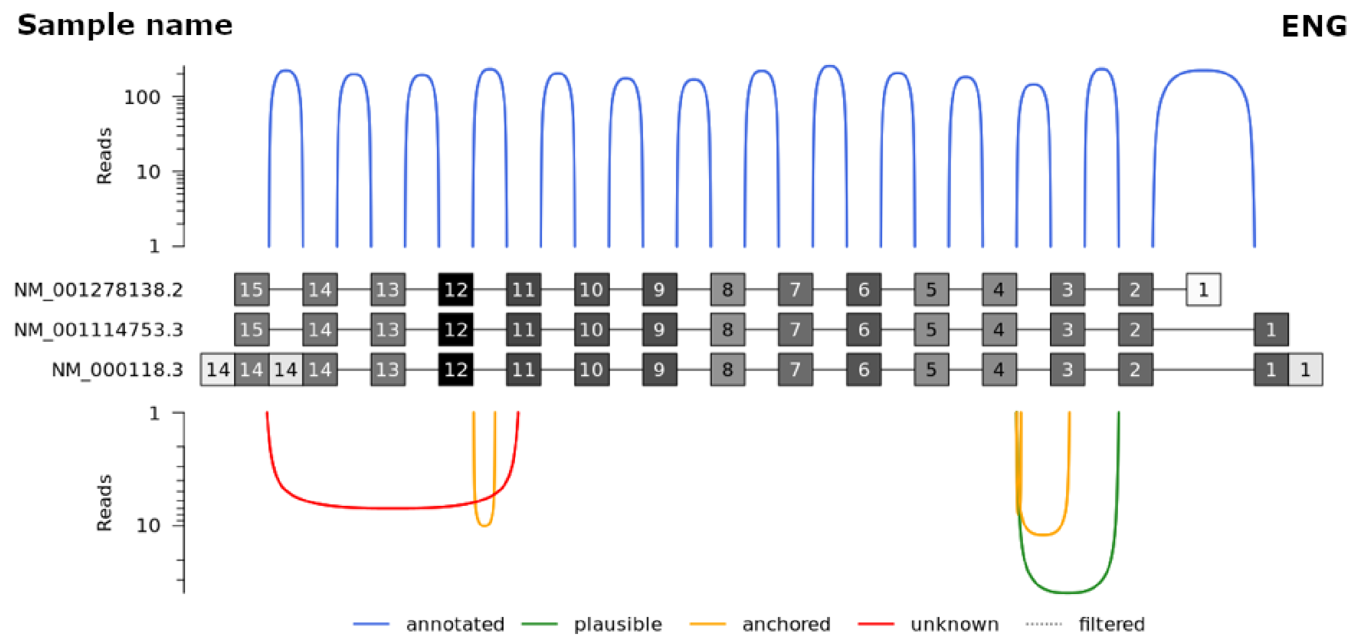

2.3 Supp Figure 3: True and False positive fusion events on increasing RNA concentration

Amount of True (plain lines) and False (dashed lines) positive fusion events detected on the six SeraSeq samples with an increasing RNA quantity, 6.25 ng, 10 ng, 12.5 ng, 25 ng, and two samples with 50 ng, containing 15 expected fusion events for SAMI (top panels) and tools from rnafusion (bottom panel). For the 50 ng quantity, the mean value of the two samples is represented. For SAMI, four thresholds have been used (from left to right): no-filter, sensitive, intermediate, and stringent. For rnafusion, the predictions of three tools are shown (from left to right): Arriba, FusionCatcher, and STAR-fusion.

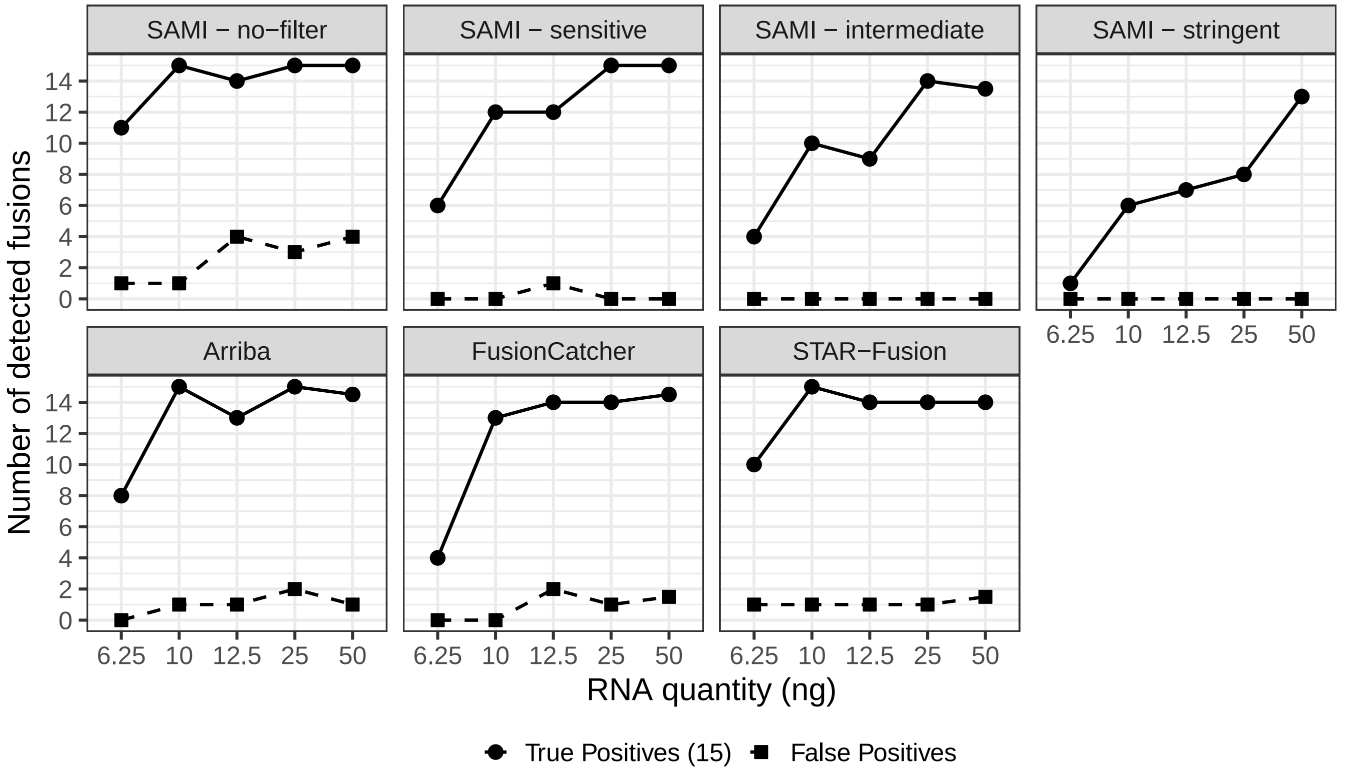

3 Supplemental Tables

3.1 Supp Table 1: Events expected in the Seraseq® sample

Human Genome Variation Society (HGVS) nomenclature of 17 aberrations expected in the Seraseq® commercial sample (adapted from https://www.seracare.com/globalassets/seracare-resources/pi-0710-0497-seraseq-fusion-rna-mix-v4.pdf). TMPRSS2-ERG was not considered as none of the two genes involved are captured by the "small" RNA-seq panel the sample was sequenced with.

event	type	HGVS
EGFR Variant III	splicing	EGFRNM_005228.5:r.350_1150del
MET ex 14 Skipping	splicing	METNM_001127500.3:r.3338_3478del
CCDC6-RET	gene fusion	CCDC6NM_005436.5:r.1_435_RETNM_020975.6:r.2327_5617
CD74-ROS1	gene fusion	CD74NM_001025159.2:r.1_812_ROS1NM_002944.2r.5757_7368
EGFR-SEPT14	gene fusion	EGFRNM_005228.5:r.1_3207_SEPT14NM_207366.3:r.1200_3752
EML4-ALK	gene fusion	EML4NM_019063.4:r.1_1763_ALKNM_004304.4:r.4125_6265
ETV6-NTRK3	gene fusion	ETV6NM_001987.4:r.1_1283_NTRK3NM_001012338.2:r.1892_3004
FGFR3-BAIAP2L1	gene fusion	FGFR3NM_000142.4:r.1_2530_BAIAP2L1NM_018842.4:r.315_3682
FGFR3-TACC3	gene fusion	FGFR3NM_000142.4:r.1_2530_TACC3NM_006342.3:r.2066_2799
KIF5B-RET	gene fusion	KIF5BNM_004521.2:r.1_3231_RETNM_020975.6:r.2070_5617
LMNA-NTRK1	gene fusion	LMNANM_170707.3:r.1_762_NTRK1NM_001012331.1:r.1290_2647
NCOA4-RET	gene fusion	NCOA4NM_001145260.1:r.1_1014_RETNM_020975.6:r.2327_5617
PAX8-PPARG	gene fusion	PAX8NM_003466.4:r.1_1253_PPARGNM_138712.3:r.246_1892
SLC34A2-ROS1	gene fusion	SLC34A2NM_006424.2:r.1_460_ROS1NM_002944.2:r.5757_7368
SLC45A3-BRAF	gene fusion	SLC45A3NM_033102.3:r.1_109_BRAFNM_004333.5:r.1206_4560
TFG-NTRK1	gene fusion	TFGNM_006070.5:r.1_851_NTRK1NM_001012331.1:r.1234_2647
TPM3-NTRK1	gene fusion	TPM3NM_153649.3:r.1_794_NTRK1NM_001012331.1:r.1234_2647
TMPRSS2-ERG	off-target	TMPRSS2NM_005656.3:r.1_78_ERGNM_004449.4:r.124_5042

3.2 Supp Table 2: Detailed computation time

CPU and wall-clock times (in hours) measured running SAMI and SpliceLauncher (SL) on three runs from two RNA-seq panels and on ASimulatoR data. Details are given for the main processes (QC, UMI, splicing, STAR). Other processes are grouped into other.

job	dataset	tool	CPU time	wall time	other	QC	UMI	splicing	STAR
671762	ASR	SAMI	162.398	3.226	10.188	27.368	0	5.493	119.349
671763	ASR	SAMI	164.522	3.18	9.792	25.585	0	5.548	123.597
671764	ASR	SAMI	162.957	3.188	9.98	28.095	0	5.42	119.462
671765	ASR	SAMI	163.158	2.917	10.159	27.796	0	5.809	119.394
671766	ASR	SAMI	162.832	3.459	10.65	27.599	0	5.503	119.08
618343	large-1	SAMI	103.21	2.25	6.729	12.075	30.919	4.432	49.056
618344	large-2	SAMI	114.211	2.531	7.782	12.774	34.323	4.759	54.573
618345	large-3	SAMI	113.777	2.555	7.946	12.646	32.168	4.688	56.33
630661	large-1	SAMI	104.955	2.615	7.095	11.566	29.933	4.49	51.872
630662	large-2	SAMI	113.77	2.764	8.104	12.896	34.122	4.716	53.932
630663	large-3	SAMI	114.163	2.454	7.835	13.144	34.603	4.633	53.948
630690	large-1	SAMI	101.202	2.184	6.778	11.474	29.558	4.475	48.917
630691	large-2	SAMI	113.016	2.531	7.82	12.752	33.915	4.741	53.789
630692	large-3	SAMI	114.336	2.547	8.063	12.757	33.948	4.716	54.852
630706	large-1	SAMI	102.431	2.178	6.847	11.836	29.121	4.275	50.352
630707	large-2	SAMI	114.287	2.417	7.571	12.992	33.338	4.681	55.704
630708	large-3	SAMI	112.823	2.733	8.225	12.871	32.807	4.722	54.198
630722	large-1	SAMI	99.647	2.204	6.839	11.771	29.765	4.519	46.752
630723	large-2	SAMI	110.482	2.353	7.691	13.164	33.972	4.48	51.175
630724	large-3	SAMI	112.648	2.474	8.007	12.727	34.351	4.682	52.882
618337	small-1	SAMI	17.629	0.59	0.85	0.807	2.082	1.825	12.065
618338	small-2	SAMI	15.728	0.446	0.619	0.526	1.529	1.125	11.929
618339	small-3	SAMI	17.516	0.518	0.706	0.667	1.867	1.422	12.853
630655	small-1	SAMI	18.964	0.636	0.925	0.885	2.162	1.888	13.105
630656	small-2	SAMI	16.17	0.507	0.697	0.618	1.599	1.348	11.908
630657	small-3	SAMI	18.004	0.521	0.734	0.783	1.894	1.349	13.243
630684	small-1	SAMI	17.477	0.549	0.787	0.827	2.098	1.839	11.927
630685	small-2	SAMI	14.521	0.433	0.593	0.531	1.539	1.318	10.54
630686	small-3	SAMI	16.23	0.507	0.684	0.703	1.857	1.502	11.484
630700	small-1	SAMI	17.702	0.593	0.832	0.82	2.076	1.887	12.087
630701	small-2	SAMI	15.5	0.449	0.594	0.551	1.528	1.328	11.5
630702	small-3	SAMI	16.761	0.504	0.681	0.697	1.845	1.452	12.086
630717	small-2	SAMI	13.699	0.415	0.559	0.517	1.628	1.126	9.868
630718	small-3	SAMI	15.874	0.479	0.664	0.697	1.804	1.331	11.377
671761	small-1	SAMI	19.235	0.591	0.851	0.82	2.001	1.781	13.783
618335	SeraSeq	SAMI	10.155	0.421	0.493	0.323	0.943	1.284	7.112
630653	SeraSeq	SAMI	11.339	0.454	0.563	0.339	0.859	1.293	8.284
630682	SeraSeq	SAMI	11.05	0.442	0.506	0.314	0.816	1.294	8.119
630698	SeraSeq	SAMI	11.63	0.453	0.526	0.326	0.927	1.294	8.557
630714	SeraSeq	SAMI	11.266	0.453	0.524	0.318	0.876	1.299	8.25
618350	ASR	SL	31.496	3.776	0	0	0	6.814	24.682
630681	ASR	SL	30.877	3.775	0	0	0	6.822	24.056
630697	ASR	SL	30.938	3.757	0	0	0	6.813	24.125
630713	ASR	SL	31.902	3.857	0	0	0	6.832	25.071
630729	ASR	SL	30.881	3.804	0	0	0	6.803	24.078
618346	large-1	SL	23.138	2.432	0	0	6.129	3.84	13.169
618347	large-2	SL	25.44	2.692	0	0	6.988	4.307	14.146
618348	large-3	SL	24.708	2.677	0	0	7.044	4.24	13.424
630664	large-1	SL	22.844	2.416	0	0	6.143	3.843	12.858
630665	large-2	SL	24.386	2.678	0	0	7	4.296	13.09
630666	large-3	SL	24.779	2.672	0	0	7.092	4.232	13.456
630693	large-1	SL	21.551	2.401	0	0	6.106	3.831	11.614
630694	large-2	SL	24.154	2.674	0	0	6.969	4.306	12.879
630695	large-3	SL	24.674	2.675	0	0	7.068	4.237	13.368
630709	large-1	SL	21.655	2.405	0	0	6.109	3.839	11.707
630710	large-2	SL	24.227	2.677	0	0	6.988	4.294	12.944
630711	large-3	SL	24.716	2.683	0	0	7.024	4.235	13.457
630725	large-1	SL	21.554	2.412	0	0	6.097	3.835	11.622
630726	large-2	SL	24.253	2.681	0	0	6.967	4.302	12.983
630727	large-3	SL	24.613	2.68	0	0	7.084	4.229	13.3
618340	small-1	SL	5.491	0.676	0	0	0.433	1.163	3.894
618341	small-2	SL	2.375	0.27	0	0	0.331	0.387	1.657
618342	small-3	SL	2.277	0.256	0	0	0.367	0.377	1.533
630658	small-1	SL	5.399	0.678	0	0	0.433	1.166	3.8
630659	small-2	SL	2.253	0.273	0	0	0.34	0.411	1.503
630660	small-3	SL	3.228	0.269	0	0	0.521	0.385	2.322
630687	small-1	SL	3.365	0.65	0	0	0.439	1.166	1.76
630688	small-2	SL	2.208	0.263	0	0	0.333	0.39	1.485
630689	small-3	SL	2.239	0.256	0	0	0.366	0.377	1.496
630703	small-1	SL	3.881	0.651	0	0	0.437	1.166	2.278
630704	small-2	SL	2.198	0.262	0	0	0.329	0.389	1.479
630705	small-3	SL	2.242	0.256	0	0	0.368	0.377	1.497
630719	small-1	SL	3.307	0.652	0	0	0.433	1.17	1.704
630720	small-2	SL	2.185	0.262	0	0	0.323	0.388	1.474
630721	small-3	SL	2.264	0.259	0	0	0.361	0.382	1.521
618336	SeraSeq	SL	2.898	0.375	0.105	0	0.178	0.52	2.096
630654	SeraSeq	SL	1.639	0.352	0.104	0	0.189	0.527	0.818
630683	SeraSeq	SL	1.636	0.349	0.106	0	0.177	0.521	0.832
630699	SeraSeq	SL	1.746	0.358	0.105	0	0.177	0.521	0.943
630715	SeraSeq	SL	1.616	0.352	0.105	0	0.179	0.527	0.806

3.3 Supp Table 3: DNA mutations of MET related to exon-skippings

Subset of the 1,658 clinical samples analyzed with both DNA-sequencing of MET splicing sites and RNA-sequencing. This table is limited to the 47 samples considered positive either at the DNA-level or at the RNA-level (or both). Columns "Position in MET" and "VAF" (Variant Allele Frequency) refer to DNA-sequencing results, while columns "left/right depth/PSI" refer to RNA-sequencing results. NI: Not Interpretable.

ID	DNA	RNA	Position in MET	VAF	left PSI	left depth	right PSI	right depth
1	Skip	Skip	c.2888-35_2888-20del	32%	50%	139	80%	86
2	Skip	Skip	c.3028G>C	7%	35%	763	52%	518
3	Skip	Skip	c.2888-22_2891del	5%	66%	925	73%	842
4	Skip	Skip	c.2888-17_2888-5del	78%	85%	7987	92%	7342
5	Skip	Skip	c.3028+3A>T	39%	87%	538	89%	526
6	Skip	Skip	c.3028+3A>G	14%	71%	143	78%	130
7	Skip	Skip	c.3028+3A>G	54%	83%	434	83%	433
8	Skip	Skip	c.3028G>T	72%	71%	3240	79%	2935
9	Skip	Skip	c.3028+3A>T	38%	83%	432	89%	404
10	Skip	Skip	c.2888-21_2888-10delinsA	13%	99%	17598	100%	17567
11	Skip	Skip	c.3028G>C	35%	90%	1426	93%	1382
12	Skip	Skip	c.3028+1G>C	14%	58%	1074	69%	906
13	Skip	Skip	c.2888-23_2888-11del	76%	94%	113	97%	109
14	Skip	Skip	c.2888-35_2888-20del	45%	96%	132	98%	129
15	Skip	Skip	c.2888-17_2888-7del	99%	99%	5228	100%	5211
16	Skip	Skip	c.2942-39_2942-19del	32%	93%	15	93%	15
17	Skip	Skip	c.3072_3082+10delinsCTTC	31%	88%	1723	92%	1653
18	Skip	Skip	c.3028+2T>C	16%	81%	245	88%	224
19	Skip	Skip	c.3082+3A>G	32%	95%	3029	97%	2961
20	Skip	Skip	c.3028+3A>G	60%	83%	2742	88%	2575
21	Skip	Skip	c.2888-21_2888-7del	34%	49%	480	58%	399
22	Skip	Skip	c.3028G>A	77%	99%	11555	99%	11465
23	Skip	Skip	c.3025_3028+1del	24%	73%	1598	81%	1425
24	Skip	Skip	c.2888-5_2904del	7%	88%	1166	89%	1158
25	Skip	Skip	c.3028G>A	17%	83%	768	90%	714
26	Skip	Skip	c.2888-28_2888-10delinsT	32%	86%	2170	90%	2070
27	Skip	Skip	c.2888-32_2888-9del	23%	95%	3369	97%	3312
28	Skip	Skip	c.2888-46_2888-22del	77%	97%	384	98%	379
29	Skip	Skip	c.2888-21_2888-10delinsCT	24%	85%	4696	91%	4389
30	Skip	Skip	c.2888-17_2888-16insAGAA	24%	91%	4091	95%	3941
31	Skip	Skip	c.3028+3A>G	53%	74%	472	78%	450
32	Skip	Skip	c.3028G>A	16%	93%	2641	95%	2586
33	Skip	Skip	c.2888-12_2888-2del	43%	92%	1760	92%	1743
34	Skip	Skip	c.3028G>T	43%	91%	401	95%	384
35	Skip	Skip	c.2888-32_2888-15del	34%	94%	62	97%	60
36	Skip	Skip	c.3007_3027del	32%	60%	57	92%	37
37	No Skip	Skip			41%	3689	53%	2851
38	No Skip	Skip			86%	36	97%	32
39	No Skip	Skip			9%	216	9%	210
40	No Skip	Skip			97%	2281	98%	2258
41	No Skip	Skip			9%	8721	14%	5504
42	No Skip	Skip			80%	235	76%	249
43	No Skip	Skip			56%	27	58%	26
44	NI	Skip			31%	184	40%	142
45	Skip	No Skip	c.2888-24_2888-23del	48%	10%	214	7%	297
46	Skip	NI	c.3028G>C	59%				
47	Skip	NI	c.3028+3A>G	27%				

Additionnal comments on discrepancies:

- Case 37: Missed out mutation c.2888-29_3028+38del, VAF=11%, depth=4262
- Case 38: Missed out mutation c.2888-35_2888-20del, VAF=48%, depth=1949
- Case 39: DNA wild-type but low PSI in RNA
- Case 40: No explanation
- Case 41: No explanation (low PSI in RNA)
- Case 42: No explanation
- Case 43: Missed out mutation c.2888-18_2888-9del, VAF=7%, depth=3907
- Case 44: Missed out mutation c.2888-18_c.2888-1del, VAF=2.8%, depth=3339
- Case 45: Only 1 bp on poly-pyrimidine tract, low PSI in RNA
- Case 46: Low quality RNA-seq
- Case 47: Low quality RNA-seq

References

[1] Alexander Dobin, Carrie A. Davis, Felix Schlesinger, Jorg Drenkow, Chris Zaleski, Sonali Jha, Philippe Batut, Mark Chaisson, and Thomas R. Gingeras. STAR: ultrafast universal RNA-seq aligner. *Bioinformatics (Oxford, England)*, 29(1):15–21, January 2013.

[2] Brian J. Haas, Alexander Dobin, Bo Li, Nicolas Stransky, Nathalie Pochet, and Aviv Regev. Accuracy assessment of fusion transcript detection via read-mapping and de novo fusion transcript assembly-based methods. *Genome Biology*, 20(1):213, October 2019.

[3] Raphaël Leman, Valentin Harter, Alexandre Atkinson, Grégoire Davy, Antoine Rousselin, Etienne Muller, Laurent Castéra, Fréderic Lemoine, Pierre de la Grange, Marine Guillaud-Bataille, Dominique Vaur, and Sophie Krieger. SpliceLauncher: a tool for detection, annotation and relative quantification of alternative junctions from RNAseq data. *Bioinformatics (Oxford, England)*, 36(5):1634–1636, March 2020.

[4] Quirin Manz, Olga Tsoy, Amit Fenn, Jan Baumbach, Uwe Völker, Markus List, and Tim Kacprowski. ASimulatoR: splice-aware RNA-Seq data simulation. *Bioinformatics (Oxford, England)*, 37(18):3008–3010, September 2021.
